# Factors affecting mortality after traumatic brain injury in a resource‐poor setting

**DOI:** 10.1002/bjs5.50243

**Published:** 2019-12-19

**Authors:** R. Okidi, D. M. Ogwang, T. R. Okello, D. Ezati, W. Kyegombe, D. Nyeko, N. J. Scolding

**Affiliations:** ^1^ Department of Surgery St Mary's Hospital Lacor Uganda; ^2^ Faculty of Medicine Gulu University Gulu Uganda; ^3^ Department of Surgery, Faculty of Medicine Lira University Lira Uganda; ^4^ Institute of Clinical Neurosciences University of Bristol Bristol UK

## Abstract

**Background:**

Traumatic brain injury (TBI) is a major cause of long‐term disability and economic loss to society. The aim of this study was to assess the factors affecting mortality after TBI in a resource‐poor setting.

**Methods:**

Chart review was performed for randomly selected patients who presented with TBI between 2013 and 2017 at St Mary's Hospital, Lacor, northern Uganda. Data collected included demographic details, time from injury to presentation, and vital signs on arrival. In‐hospital management and mortality were recorded. Severe head injury was defined as a Glasgow Coma Scale score below 9.

**Results:**

A total of 194 patient charts were reviewed. Median age at time of injury was 27 (i.q.r. 2–68) years. The majority of patients were male (M : F ratio 4·9 : 1). Some 30·9 per cent of patients had severe head injury, and an associated skull fracture was observed in 8·8 per cent. Treatment was mainly conservative in 94·8 per cent of patients; three patients (1·5 per cent) had burr‐holes, four (2·1 per cent) had a craniotomy, and three (1·5 per cent) had skull fracture elevation. The mortality rate was 33·0 per cent; 46 (72 per cent) of the 64 patients who died had severe head injury. Of the ten surgically treated patients, seven died, including all three patients who had a burr‐hole. In multivariable analysis, factors associated with mortality were mean arterial pressure (*P* = 0·012), referral status (*P* = 0·001), respiratory distress (*P* = 0·040), severe head injury (*P* = 0·011) and pupil reactivity (*P* = 0·011).

**Conclusion:**

TBI in a resource‐poor setting remains a major challenge and affects mainly young males. Decisions concerning surgical intervention are compromised by the lack of both CT and intracranial pressure monitoring, with consequent poor outcomes.

## Graphical abstract

Traumatic brain injury (TBI) in 194 patients in a resource‐poor setting was analysed. Some 30·9 per cent had severe TBI, and associated skull fracture was observed in 8·8 per cent. Treatments offered were mainly conservative. The mortality rate was 33·0 per cent, with 72 per cent resulting from severe head injury. Seven of ten surgically treated patients died, including all three patients who had received a burr‐hole. Factors associated with mortality in multivariable analysis were mean arterial pressure, referral status, respiratory distress, severe head injury and pupil reactivity. TBI remains a major challenge in resource‐poor settings, with the younger male population mainly involved. Decisions regarding interventions are greatly compromised by the lack of CT and intracranial pressure monitoring.

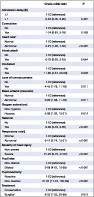

Mortality remains high

## Abstract (es)

**Antecedentes:**

La lesión cerebral traumática (*traumatic brain injury*, TBI) es un insulto al cerebro causado por una fuerza física externa que produce un estado de conciencia disminuido o alterado, lo que resulta en un deterioro de las capacidades cognitivas o del funcionamiento físico. Es una causa importante de discapacidad a largo plazo y pérdida económica para la sociedad. El objetivo de este estudio fue evaluar los factores que afectan a la mortalidad después de una TBI en un entorno de escasos recursos.

**Métodos:**

Se realizó la revisión de historias clínicas de pacientes seleccionados al azar que habían presentado una TBI entre 2013 y 2017 en el Hospital St. Mary's, un hospital privado sin ánimo de lucro ubicado en el distrito de Gulu, Lacor, en el norte de Uganda. Se recogieron datos de las características demográficas, intervalo de tiempo entre la lesión y la atención médica, y signos vitales a la llegada al hospital. Se registró también el manejo hospitalario y la mortalidad. El traumatismo craneal grave se definió como aquel con una escala de coma de Glasgow (*Glasgow Coma Scale*, GCS) por debajo de 9.

**Resultados:**

Se revisaron 194 historias clínicas de pacientes. La mediana de edad en el momento del traumatismo fue de 27 (rango intercuartílico de 2 a 68) años. La mayoría eran varones con una relación varón:mujer de 4,9:1. En el 38,1% de los casos los traumatismos craneales fueron calificados como graves y se observó una fractura de cráneo asociada en el 8,8% de los pacientes. Los tratamientos ofrecidos fueron principalmente conservadores en el 94,9%; tres pacientes (1,6%) precisaron trépanos, en cuatro pacientes (2,1%) se realizó una craneotomía y otros tres pacientes (1,6%) precisaron elevación de una fractura craneal con hundimiento. La mortalidad fue del 33,0%; El 71,9% de ellos tenían un traumatismo craneal grave. Entre los pacientes tratados quirúrgicamente, siete (70%) murieron, incluidos los tres pacientes en los que se realizó un trépano. Los factores asociados con la mortalidad en el análisis multivariable fueron la presión arterial media (*P* < 0,05), el estado en el traslado (*P* < 0,05), la dificultad respiratoria (*P* = 0,040), el traumatismo craneal grave (*P* = 0,012) y la reactividad pupilar (*P* = 0,011).

**Conclusión:**

El TBI en un entorno con pocos cursos continúa siendo un desafío importante, afectando principalmente a varones jóvenes. Las decisiones relativas a la intervención quirúrgica y el momento de su práctica están seriamente comprometidas por la falta de disponibilidad de tomografía computarizada (TAC) y monitorización de la presión intracraneal, lo que conlleva unos pobres resultados.

## Introduction

Traumatic brain injury (TBI) is an insult to the brain, not degenerative or congenital in nature, but caused by an external physical force, that may produce a diminished or altered state of consciousness, resulting in an impairment of cognitive ability or physical functioning^1^. TBI is an important public health problem worldwide^2^. It is a major cause of long‐term disability and economic loss to society^3^. In the USA alone, approximately 1·7 million TBIs occur each year, either in isolation or as part of polytrauma. TBI remains the highest global cause of morbidity and mortality^4^.

Classifications of TBI vary; however, the Glasgow Coma Scale (GCS) is the commonest and most widely used system for classifying TBI severity at the time of injury. The GCS is a 3–15‐point scale, assessing the patient's conscious level and neurological functioning^5^. A GCS score of 13–15 is defined as mild, 9–12 as moderate, and 3–8 as severe.

Much of the neurological damage resulting from TBI does not occur immediately but in the minutes, hours and days that follow^6^. The primary injury is due to irreversible mechanical injury, but the resulting cerebral oedema contributes to secondary injury from raised intracranial pressure, hypotension, hypoxia, anaemia, seizures, hypoglycaemia and hyperthermia. Therefore, prevention and correct management of this complication improves outcome from head injury, and urgent treatment of head‐injured patients is of utmost importance.

In Africa, there is wide variation in TBI epidemiology^7^. The majority of TBI occurs in young patients aged 19–40 years, and mainly affects males^8^. TBI is a major cause of mortality and morbidity in sub‐Saharan Africa. In Mulago National Referral Hospital in the Ugandan capital Kampala, TBI had an overall mortality rate of 9·6 per cent, ranging from 4·7 per cent for mild and moderate to 55 per cent for severe head injuries^9^.

TBI in sub‐Saharan Africa has been poorly studied, particularly in non‐metropolitan areas and in hospital centres that lack the imaging facilities found in large city hospitals. St Mary's Hospital, Lacor, located in northern Uganda along Gulu–Juba highway, is a centre that receives a high number of injured patients with associated TBI. It is limited in its capability for neurosurgical care, with no CT available, and death from TBI remains high, second only to sepsis as the overall cause of death in the hospital in 2017. A 2017 study^10^ of all admissions to the ICU in St Mary's Hospital found the mortality rate amongst patients with severe TBI to be 16·3 per cent, but the causes were not explored.

Determining the mortality risk factors in patients with TBI would enable the healthcare provider to carry out correct and early allocation of resources to help reduce the morbidity and mortality rate, and would be of great benefit in resource‐limited settings. The aim of this study was to assess the current scale of the problem, and to determine factors associated with mortality among patients presenting with TBI to St Mary's Hospital, Lacor.

## Methods

A chart review of patients admitted to St Mary's Hospital, Lacor, with a diagnosis of TBI during 2013–2017 was undertaken. The study was approved by the hospital's institutional research and ethics committee (LHIREC). Given the retrospective and notes‐based nature of the study, the LHIREC board waived the need to gain consent from individual patients.

St Mary's Hospital, Lacor, is a private, not‐for‐profit, church hospital situated in Gulu District, northern Uganda. The hospital has significant overseas support, and patient care is subsidized in order to fulfil its mission of serving the poorest patients to the highest standards possible. The hospital is also a university teaching hospital for the Government University of Gulu, Faculty of Medicine. Gulu Regional Referral Hospital is about 3 miles away, and also receives casualties. It is likely that the patient population in the present study is representative of the population in the region. Neither cost nor religious considerations are likely to influence initiating a transfer to St Mary's Hospital.

The sample size required was calculated by Slovin's formula as 194 patients (*Appendix* *S1*, supporting information). All files in the hospital archives for the Department of Surgery with a diagnosis of trauma were selected and sorted for associated TBI. The 194 patient charts were selected randomly, and data were extracted from the sampled files using predesigned semistructured questionnaires. Data were collected on age, sex, marital status, referral status, delay before arrival to hospital, degree of TBI severity, pupil size and reactivity to light, systolic BP, pulse rate, respiratory rate, intubation status and oxygen saturation (*Appendix* *S2*, supporting information). In‐hospital management and mortality were recorded. All collected data were transferred into a computerized database created using Epi Info™ 7.0 (https://www.cdc.gov/epiinfo).

### Statistical analysis

Statistical analysis was performed using statistical software STATA® version 13.0 (StataCorp, College Station, Texas, USA). Univariable analysis was done in which the sociodemographic factors were described.

Mean(s.d.) values were calculated for continuous variables, and proportion and frequency tables were used to summarize categorical variables. Continuous variables were categorized, then bivariable and multivariable logistic regression analyses were performed for mortality factors, calculating the crude and adjusted odds ratios (ORs), and *P* values. *P* < 0·050 was considered statistically significant.

## Results

A total of 375 patients were treated for TBI during the study period. The charts of 194 patients were reviewed; their baseline characteristics are shown in *Table* 1.

**Table 1:** Baseline characteristics and vital observations on arrival

	No. of patients (*n* = 194)
**Age (years)**	
< 65	186 (95·9)
≥ 65	8 (4·1)
**Sex**	
M	161 (83·0)
F	33 (17·0)
**Marital status**	
Married	100 (51·5)
Single	94 (48·5)
**Referral status**	
Referral	94 (48·5)
Non‐referral	100 (51·5)
**Delay before admission (h)**	*n* = 158
≤ 1	9 (5·7)
> 1	149 (94·3)
**GCS score** *	
> 13	74 (38·1)
9–13	60 (30·9)
< 9	60 (30·9)
**Pupil size**	
Normal	135 (69·6)
Anisocoria	35 (18·0)
Bilateral dilation	24 (12·4)
**Pupil reactivity**	
Normal	150 (77·3)
Sluggish	30 (15·5)
Non‐reactive	14 (7·2)
**Mean systolic BP (mmHg)**	*n* = 154
< 90	6 (3·9)
90–139	124 (80·5)
≥ 140	24 (15·6)
**Pulse rate (beats/min)**	*n* = 172
≤ 60	12 (7·0)
61–100	115 (66·9)
> 100	45 (26·2)
**Respiratory rate (breaths/min)**	*n* = 173
< 12	0 (0)
12–20	145 (83·8)
> 20	28 (16·2)
**Oxygen saturation (%)**	*n* = 185
< 90	18 (9·7)
≥ 90	167 (90·3)

Values in parentheses are percentages.

* Definition of severity of traumatic brain injury (TBI): severe TBI, Glasgow Coma Scale (GCS) score above 13; moderate TBI, GCS score 9–13; mild TBI, GCS score below 9.

A variety of causes of TBI were identified. Notably, 62 individuals (32·0 per cent) came off their motorcycle and 54 (27·8 per cent) were hit by a motorcycle; other motor vehicles hit a further 33 individuals (17·0 per cent). Assault was the cause of TBI in 37 patients (19·1 per cent), and eight (4·1 per cent) fell from a height. Some 161 patients (83·0 per cent) were males, and 129 (66·5 per cent) were in the reproductive age group. Overall, 155 patients (79·9 per cent) were under 40 years of age. All injuries were blunt rather than penetrating.

Patients with head injury presented to the accident and emergency department with varying GCS scores and varying duration from the time of injury. The GCS score ranged from 3 to 15 (mean(s.d.) 10(3)). Some 94·3 per cent of the patients (149 of 158) presented to the hospital more than 24 h from the time of injury. Seventy‐four patients (38·1 per cent) presented with mild, 60 (30·9 per cent) with moderate and 60 (30·9 per cent) with severe TBI. Twenty‐four (12·4 per cent) of the patients were admitted with bilaterally dilated pupils and 35 (18·0 per cent) had anisocoria. Thirty‐two patients (16·5 per cent) had seizure episodes, and 9·7 per cent (18 of 185) were hypoxic on admission.

Plain skull X‐ray evaluation for possible skull fracture was performed in 37 patients (19·1 per cent) admitted with a head injury. Skull X‐ray was performed at the doctor's discretion on clinical suspicion of skull fracture for those not undergoing ventilation. However, for those under ventilation, transferring the patient to the radiology department for emergency skull X‐ray was often not feasible until stabilized. Of those X‐rayed, 17 (46 per cent) had skull fractures: nine (24 per cent) depressed skull fractures and eight (22 per cent) linear fractures. Other associated injuries were: soft tissue injury in all patients, blunt chest trauma in nine (4·6 per cent), long bone fractures in 11 (5·7 per cent), oromaxillofacial injury in 15 (7·7 per cent) and visceral injury in two (1·0 per cent). No patient had associated spine or spinal cord injury at presentation.

Treatments offered were conservative and surgical. Some 184 patients (94·8 per cent) were treated conservatively, three (1·5 per cent) had burr‐holes, four (2·1 per cent) had a craniotomy and three (1·5 per cent) had skull elevation performed.

The mortality rate was 33·0 per cent (64 of 194), with 56 deaths (88 per cent of total deaths) in male patients. Seven of the ten surgically treated patients died, and 57 (31·0 per cent) of the 184 patients managed conservatively. All three patients who had burr‐holes performed died.

### Factors associated with mortality

The mortality rate varied depending on the GCS score of the patient at presentation (adjusted OR 10·30, 95 per cent c.i. 1·94 to 54·70; *P* = 0·012) (*Table* 2). The highest mortality was in patients with severe TBI, contributing 72 per cent of the deaths (46 of 64), with mild TBI contributing least (1 of 64, 2 per cent).

**Table 2:** Multivariable logistic regression analysis of mortality factors

	Adjusted odds ratio	*P*
Heart rate	1·00 (0·20, 5·07)	0·976
Intubation	4·76 (0·17, 131·91)	0·357
Loss of consciousness	1·18 (0·16, 9·49)	0·841
Mean arterial pressure	16·81 (2·64, 106·36)	0·012
Oxygen saturation	0·13 (0·01, 1·83)	0·133
Referral	8·56 (1·99, 36·82)	0·001
Respiratory rate	11·53 (1·07, 123·10)	0·040
Severity of head injury	10·30 (1·94, 54·69)	0·011
Pupil size	0·34 (0·03, 3·43)	0·353
Pupil reactivity	65·49 (2·67, 1606·59)	0·011
Treatment	0·11 (0·02, 10·81)	0·142

Values in parentheses are 95 per cent confidence intervals.

Only eight patients (4·1 per cent) were aged 65 years or above; three of these sustained severe TBI. Mortality was not found to be related to advanced age (adjusted OR 2·10, 95 per cent c.i. 0·51 to 8·68; *P =* 0·306).

Abnormal pupillary reactivity was a mortality factor (adjusted OR 65·49, 95 per cent c.i. 2·67 to 1606·59; *P =* 0·011) (*Table* 2), and abnormal pupillary size was a good individual predictor (crude OR 9·68, 4·81 to 19·65; *P* < 0·001) (*Table* 3). However, abnormal pupillary size was a confounder in the multivariable logistic model (adjusted OR 0·34, 0·03 to 3·43; *P =* 0·353).

**Table 3:** Bivariable analysis of mortality factors

	Crude odds ratio	*P*
**Admission delay (h)**		
≤ 1	1·00 (reference)	
> 1	0·54 (0·06, 3·95)	0·491
**Convulsion**		
No	1·00 (reference)	
Yes	1·74 (0·81, 3·78)	0·163
**Heart rate** *		
Normal	1·00 (reference)	
Abnormal	3·91 (1·99, 7·62)	< 0·001
**Intoxicated** †		
No	1·00 (reference)	
Yes	0·79 (0·29, 2·15)	0·654
**Intubated**		
No	1·00 (reference)	
Yes	11·85 (2·51, 55·90)	0·022
**Loss of consciousness**		
No	1·00 (reference)	
Yes	4·45 (1·78, 11·16)	0·011
**Mean arterial pressure** ‡		
Normal	1·00 (reference)	
Abnormal	3·89 (1·38, 11·10)	0·011
**Oxygen saturation** §		
Non‐hypoxic	1·00 (reference)	
Hypoxic	5·38 (1·92, 15·31)	0·012
**Referral**		
No	1·00 (reference)	
Yes	6·98 (3·48, 3·99)	< 0·001
**Respiratory rate** ¶		
Normal	1·00 (reference)	
Abnormal	6·47 (2·74, 15·31)	< 0·001
**Severity of head injury**		
Non‐severe	1·00 (reference)	
Severe	21·23 (9·73, 47·07)	< 0·001
**Pupil size**		
Non‐dilated	1·00 (reference)	
Dilated	9·68 (4·81, 19·65)	< 0·001
**Pupil reactivity**		
Reactive	1·00 (reference)	
Non‐reactive	51·91 (17·19, 160·39)	< 0·001
**Treatment**		
Conservative	1·00 (reference)	
Surgical	6·05 (1·55, 23·65)	0·012

Values in parentheses are 95 per cent confidence intervals.

* Normal range 60–100 beats/min;

† involved alcohol exclusively;

‡ normal range 70–100 mmHg;

§ normal range 95–100 per cent;

¶ normal rate 12–20 breaths/min.

In this study, 12 (67%) of the 18 hypoxic patients died and hypoxia at admission was found to be associated with mortality (crude OR 5·38, 95 per cent c.i. 1·92 to 15·31; *P =* 0·012) (*Table* 3). However, hypoxia was a confounding factor in the multivariable logistic model (adjusted OR 0·13, 0·01 to 1·83; *P =* 0·133) (*Table* 2). Respiratory distress was associated with mortality (adjusted OR 11·53, 1·07 to 123·10; *P =* 0·040). Hypotension contributed to mortality in only four (6 percent) of the 64 patients who died and was not a significant factor affecting mortality (crude OR 4·13, 0·71 to 23·02; *P =* 0·124).

Eighty‐eight patients were transferred from other hospitals or health centres. The largest single origin of transfer (20 patients, 23 per cent) was from Gulu Regional Referral Hospital, just over 3 miles away. However, all other transfers were from facilities considerably more distant, most from around 25 miles, but also including nine patients (10 per cent of the 88 transferred) from Kiryandongo Hospital, 63 miles away. Fifty patients referred from other facilities died (78 per cent of the 64 who died), and referral was a strong predictor of mortality (adjusted OR 8·56, 95 per cent c.i. 1·99 to 36·82; *P =* 0·001) (*Table* 2). This could be because the 94 patients with TBI (48·5 per cent) who were referred from another health facility had already suffered secondary brain damage before accessing care. An additional aspect was the delay inevitably involved in referral and transfer (median delay 5 (i.q.r. 1–168) h).

Significant factors emerging from the bivariable logistic analysis (*Table* 3) were tested for significance in the multivariable model (*Table* 2). Mean arterial pressure, referral status, respiratory distress, severe head injury and pupillary reactivity were significant for in‐hospital mortality, whereas abnormal heart rate, intubation, loss of consciousness, oxygen saturation and surgical treatment were confounders.

## Discussion

Motor vehicle crashes and falls have been documented as the leading causes of closed brain injury, with gunshot wounds the leading cause of penetrating brain injury^11^. In the present study, the majority of TBI were caused by motor vehicle crashes, with nearly 60 per cent involving motorcycles. This result reflects the lower social economy of the environment of individuals in this study, as the majority use a motorcycle as a means of mobility.

In a study^8^ of all neurosurgical conditions conducted in southern Uganda, it was found that 76·1 per cent of TBI was in younger individuals aged 19–40 years, mainly men. The findings of the present study were similar, with 79·9 per cent of TBI occurring in people under 40 years of age, predominantly males. This finding could be due to the fact that most males in this region are unemployed; they ride motorcycles (boda‐bodas) to earn a living.

The overall mortality rate following TBI in this study was 33·0 per cent, in contrast to the overall mortality rate of 9·6 per cent found in Mulago National Referral Hospital, Kampala^9^. The results should be compared with some caution, however, as the Mulago study included only those patients referred to the neurosurgical department, whereas the present study included all patients admitted to the hospital with TBI. Nonetheless, the mortality is clearly high in the setting of this study, possibly explained by the absence of diagnostic capabilities for intracranial injury, the absence of specialist neurosurgical teams, and the long distance to the nearest neurosurgical centre.

Factors associated with mortality after TBI were explored. The higher mortality rate with more severe head injuries is as expected. More severe damage to the brain at impact plainly contributes, but other likely factors include delayed admission to hospital and lack of investigative modalities. This finding is consistent with results of a meta‐analysis highlighting the GCS score as a predictor of mortality in patients with TBI^7^, ^12^. Notably, the rates in the present study are higher than those from Mulago National Referral Hospital^9^, which has CT facilities; for those with severe head injury the Mulago mortality rate was 55 per cent, compared with 77 per cent (46 of 60) in the present study.

The lack of effect of age on mortality contrasted with a study^12^ that found increased patient age was associated with an increase in mortality and morbidity based on the Glasgow Outcome Scale. This discrepancy may be due to the limited number of elderly patients in the present study, reducing their influence on overall mortality. The findings of pupillary reactivity as an associated mortality factor are concordant with a study from a university hospital in Turkey^13^.

The findings in the present study regarding hypoxia, respiratory distress and hypotension contrast with other work^14^ showing that hypoxia and hypotension, either before or on admission, were strongly associated with outcome and could be used to predict outcomes (mortality and morbidity) of head injury. This difference could be due to the limited number of deaths to which hypotension and hypoxia contributed in the present study.

Other studies have confirmed that the extent of brain injury, assessed by CT, has a positive correlation with mortality^15^. CT provides an objective and invaluable evaluation of structural brain damage following head injury. The relative scarcity of CT in most resource‐limited settings has had a negative impact on the outcome of patients sustaining TBI^16^. The centre involved in the present study has no CT scanner, and so the extent of the structural brain damage was not known for any patient with TBI. This may have had a large influence on the decision regarding timely intervention, and hence on mortality.

As a consequence of this study, and specifically the fact that all three patients who had a burr‐hole died, a local moratorium on conducting burr‐holes has been instigated, at least until details for these patients have been examined comprehensively and clear local guidelines established.

This study has a number of limitations, including that it is retrospective and uncontrolled. Biases could also have been caused by more wealthy victims of crashes being taken by carers to private facilities in the capital, Kampala. Additionally, victims who died at the crash site may not have been brought to the hospital and so would not have been included. Within the hospital, some items of data were not collected during the patient journey and, as for all retrospective studies, there is a dependency on accurate record‐keeping. The retrospective nature also allows little or no control over co‐variables and potential confounders. The presence of a relatively close government hospital (Gulu Regional Referral Hospital) may also have introduced bias, although it is believed that patients would be brought to Lacor largely on proximity criteria. There are, however, very few other studies of outcomes from TBI in rural low‐income countries. Studies such as this provide a supporting argument for a CT scanner locally, to aid decision‐making for prompt surgical intervention and patient transfer, as TBI in rural resource‐poor settings currently remains a major challenge, with poor outcomes.

## Supporting information


**Appendix S1.** Calculation of study population
**Appendix S2.** Questionnaire for patients with head injury
