# Supplementary material for: Factors affecting mortality after traumatic brain injury in a resource‐poor setting
Source: BJS Open. 2019 Dec 19;4(2):320–5. doi: 10.1002/bjs5.50243 (PMC7093795; doi:10.1002/bjs5.50243)
Supplement: Supplementary file 1 — Appendix S1. Calculation of study population Appendix S2. Questionnaire for patients with head injury [file BJS5-4-320-s001.docx]

**BJS5_50243**

**Factors affecting mortality after traumatic brain injury in a resource-poor setting**

**R. Okidi, D. M. Ogwang, T. R. Okello, D. Ezati, W. Kyegombe, D. Nyeko and N. J. Scolding**

## Appendix S1 Calculation of study population

The population to study comprised all patients admitted with a diagnosis of traumatic brain injury during the study period. We studied a sample of these, the size of which (n) was calculated using Slovin’s Formula.

Therefore, $n=N/(1+\mathrm{Ne}^{2})$

Where n is the required sample size, N is the population of Traumatic Brain Injury (TBI) patients managed at St. Mary’s Hospital Lacor over 5 years (2013 - 2017).

In this study, we estimated the allowable error margin (e) at 5%. This, therefore, gave us a confidence interval of 95%.

$n=375/(1+{375(0.05}^{2})$,

By computing, $n=193.548$

$$n=194$$

**Appendix S2** Questionnaire for patients with head injury

## Semi structured questionnaire for head injury patients in St. Marys Hospital-Lacor

*Please choose the correct details for the patient.*

**1.Fill in the patient’s socio-demographic details**

Date of admission: *Year /month / day*,

Patient hospital number: PLH: __________________

Date of birth: *Year /month / day*,

Sex: Male Female

Place of residence: ________________________________

| Marital status | Single |  |
| --- | --- | --- |
|  | Married |  |
|  | Divorced |  |
| Level of education | No formal education |  |
|  | Primary |  |
|  | Secondary |  |
|  | Tertiary institution |  |
|  | University |  |
| Employment status | Not employed |  |
|  | Employed |  |
| Referral status | Not a Referral |  |
|  | Referral |  |

What’s the time duration in hours from the time of injury to arrival to the accident & emergency department of St. Marys Hospital-Lacor? …………….. (Hours)

What was the referral center? ………………………………

If he/she is a referral, how far (miles) is the referral center from St. Marys Hospital-Lacor? ……………….

**2.This section captures the patients; clinical details upon arrival to the accident and emergency department of St. Marys Hospital-Lacor**

**Primary survey assessment**

1. Is the airway (A) obstructed? Yes/ No
2. What’s the respiratory rate (breaths per minute)? ………………
3. Was oxygen administered to the patient? Yes / No
   1. If yes, what was the mode of delivery? Nasal prong/ rebreather face mask/ non-rebreather facemask
4. What’s the blood pressure at admission? Systolic _____ mmHg, Dystolic _____ mmHg
5. What’s the pulse rate? ______ (Beats per minute).
6. Did he/she smell alcohol? Yes / No
7. Was there loss of consciousness? Yes / No
8. Did he convulse? Yes / No
9. GCS: Eye opening ___/4, Best verbal response ____/5, Best motor response ____/6
10. Measurement of pupil sizes: left ……… (mm), right ………… (mm)
11. How did the pupils react to light? Reacted / sluggishly reacted / fixed
12. What’s the motor power?

Left upper limb ……… /5, right upper limb. ……/5

Left lower limb. ………/5, right upper limb ……/ 5

1. Was there an identifiable dermatome loss of sensation? No / Yes

If yes, what was the level? ………….

1. Was there cerebrospinal fluid (CSF) leak through the nostrils of ears? Yes / No
2. Choose the pattern of skull fracture sustained if any? Linear / stellate / depressed.
3. What was the pattern of scalp injury sustained by the patient? Abrasion/Laceration/ Avulsion.
4. What was the mechanism of the injury? Fall from height / Hit by Car/ Hit by a motorcycle/ Assaulted
5. Choose the associated injuries sustained during the trauma. Chest: Pneumothorax / Hemothorax, Abdomen: Hemoperitoneum, Pelvis: fracture

**3. This is a section on investigations done for the patient**

Sodium: _________

Chloride: _________

Potassium: _______

Random blood sugar: _____mg/dl

| Operative |  |
| --- | --- |
| Conservative |  |

**4.Choose which treatment was offered to the patient at Lacor Hospital?**

1. If surgical, when was the operation performed? Date: ______/_____/_______.
2. What operation was done?

Burr holes/ Craniotomy / Decompressive craniectomy/ Fracture elevation.

1. How many days was the patient intubated and ventilated?..........................
2. Date of discharge: _____/____/_____
3. Discharged status: Dead/ Alive
